# Many-eyes and sentinels in selfish and cooperative groups

**DOI:** 10.1073/pnas.2536017123

**Published:** 2026-08-10

**Authors:** Charlie Pilgrim, Andrew M. Bate, Anna Sigalou, Mélisande Aellen, Joe Morford, Elizabeth Warren, Christopher Krupenye, Dora Biro, Richard P. Mann

**Affiliations:** ^a^ Department of Statistics, School of Mathematics, University of Leeds, Leeds LS2 9JT, United Kingdom; ^b^ Dairy Research Institute of Asturias, Spanish National Research Council (IPLA-CSIC), Oviedo 33011, Spain; ^c^ Department of Brain and Cognitive Sciences, University of Rochester, Rochester, NY 14627; ^d^ Department of Psychological and Brain Sciences, Johns Hopkins University, Baltimore, MD 21218

**Keywords:** collective vigilance, collective intelligence, cooperation, behavioral ecology, evolution

## Abstract

Collective vigilance describes how animals in groups benefit from the predator detection efforts of others. Empirical observations typically find either a many-eyes strategy with all (or many) group members maintaining a low level of individual vigilance, or a sentinel strategy with one (or a few) individuals maintaining a high level of individual vigilance while others do not. With an analytical treatment that makes minimal assumptions, we show that these two strategies are alternative solutions to the same adaptive problem of balancing the costs of predation and vigilance. Which strategy is preferred depends on how costs scale with the level of individual vigilance: Many-eyes strategies are preferred where costs of vigilance rise gently at low levels but become steeper at higher levels (convex; e.g., an open field); sentinel strategies are preferred where costs of vigilance rise steeply at low levels and then flatten out (concave; e.g., environments with vantage points). This same dichotomy emerges whether individuals act selfishly to optimize their own fitness or cooperatively to optimize group fitness. The model is extended to explain discrete behavioral switching between strategies, differential levels of vigilance such as edge effects, and turn-taking.

## Significance

Animal groups detect predators using either a many-eyes strategy (all members maintain a low-level vigilance) or a sentinel strategy (dedicated lookouts). Despite extensive research, we lack a framework explaining when each strategy should emerge. Here, we present a model that shows how both strategies are solutions to a tradeoff between the benefits of detecting predators and the costs of keeping a lookout. The preferred solution depends on how costs scale with effort: sentinels when costs start steep but then flatten out (e.g., environments with a vantage point) and many-eyes when costs gradually steepen (e.g., open habitats). The same pattern emerges whether groups are selfish or cooperative, and points to a broader principle spanning sensory systems in biology, society, and technology.

Animals living in groups benefit from the vigilance efforts of others, whereby if one individual spots a predator approach then others in the group are alerted (1, 2). However, not all groups use the same strategy to achieve these benefits. In some species, we see “many-eyes” behavior, where numerous individuals keep a low-level lookout to collectively generate a high chance of detecting predators (3–12). In other cases we see sentinel behavior, where one or a few individuals maintain high vigilance while others forage (13–21). Despite decades of theoretical and empirical research, we lack a unified framework explaining the ecological conditions that favor each strategy (22).

Many-eyes behavior is widespread and has been observed in varied taxa spanning birds (3–7), mammals (5, 8–11), fish (12), and insects (23). Enhanced predator detection is considered a key benefit of living in groups, with the“many-eyes hypothesis” (1, 24, 25) predicting empirical patterns that larger groups enjoy a greater collective chance of detecting predators (3, 6, 7, 11, 12) while incurring lower individual costs of vigilance (3–5, 8–10). For example, yellow-eyed juncos (*Junco phaeonotus*) forage in groups on the ground for seeds while occasionally scanning for predators such as hawks, raising alarm calls upon detection (26, 27).

Sentinel behavior, while not as prevalent as many-eyes behavior, is taxonomically widespread and observed in birds (13–15), mammals (16–20), fish (21), and insects (28). Sentinels typically take up an elevated position (13, 14, 16, 19, 20, 22, 29, 30) and produce alarm calls upon detecting threats (14, 19, 29, 31). For example, in meerkats (*Suricata suricatta*), sentinels stand upright on a mound or tree while the rest of the group forage for food (16, 18, 32). Conditions that are thought to support sentinel behavior include habitats with good vantage points (19, 22, 29), the ability to coordinate (13, 14, 19, 22, 33), safe sentinel positions (26, 29, 34), mechanisms for cooperation (13, 20, 26, 35), high predation risk (16, 19, 29, 33), and resource abundance (14, 16, 22). A feature of sentinel systems is turn-taking, with individuals rotating sentinel duty over time (16, 22, 34, 36). Turn-taking requires both distinct roles (vigilance concentrated in one or a few individuals while others are not vigilant) and coordination of which members take on these roles over time. Our focus is on the conditions that favor distinct high-vigilance roles, which is a necessary condition for turn-taking.

Collective vigilance creates a social dilemma: The benefits of predator detection are shared throughout the group while costs are paid individually, generating incentives to free-ride by reducing one’s own individual vigilance while benefiting from the vigilance efforts of others (26). Game-theoretic models demonstrate that many-eyes behavior can arise in selfish groups, yet empirical observations have been interpreted as either selfish or cooperative depending on the underlying assumptions (26, 37, 38). Early work on sentinel behavior interpreted it as cooperative within a kin-selection framework (36, 39), but later models showed that sentinels can be favored by pure self-interest if sentinel positions are safer than foraging (29, 34). Empirical evidence is mixed here too, with some support for “safe selfish sentinels” (16), while other work finds evidence for cooperative dynamics (19, 20, 22, 32, 35, 39).

Existing theoretical work has typically treated many-eyes and sentinel behaviors as distinct phenomena. Influential many-eyes models focus on how larger group size enhances predator detection even while individual vigilance is lower, and how vigilance is generally expected to be higher in cooperative than selfish groups (1, 26, 37, 38). In contrast, influential sentinel models focus on how groups manage coordination through distinct signals when on guard, and how sentinels can emerge even in selfish groups when sentinels occupy safer positions than nonsentinels (13, 29, 34). Each framework captures only a subset of ecological conditions and behavior with fixed behavioral rules or specific cost–benefit functions. Consequently, the theoretical literature is fragmented and resists synthesis, with a recent review highlighting “large gaps in knowledge” (22) in the social and environmental conditions favoring sentinel over many-eyes strategies.

Here we present an analysis of how ecological conditions map onto optimal vigilance strategies, finding a clear dichotomy between either a many-eyes style strategy with vigilance spread throughout the group or a sentinel style strategy with vigilance concentrated in one (or a few) individuals. Which is preferred depends on how the individual costs of vigilance scale. In uniform habitats (e.g. open fields), vigilance costs are convex: Low levels of vigilance are cheap but costs are steepening as individuals sacrifice more foraging time at higher levels of vigilance, favoring a strategy of distributing vigilance throughout the many-eyes of the group. In structured habitats with vantage points (e.g. trees or mounds) vigilance costs are concave: It costs a lot to reach a vantage point but increasing vigilance once there is cheap, favoring the concentration of vigilance in sentinels. This same dichotomy emerges in both selfish and cooperative groups. We demonstrate this with analytical results, supported by simulations. We then extend the model to explain behavioral switching between strategies (16), differential vigilance such as edge effects (5, 40, 41), and turn-taking (16, 22, 34, 36), before discussing implications for the convergent evolution of collective vigilance. Our approach is to use a minimal model that generates a wide range of qualitative behaviors; a limitation of this approach is that it does not include realistic assumptions present in more specific models, which we explore in *Discussion*.

## 1. A Model of Collective Vigilance

The adaptive challenge of collective vigilance is balancing the threat of predation with the costs of vigilance (26, 27, 29, 30, 37, 42–45). Considering this challenge, we write down a minimal model of collective vigilance. The net fitness of individual i is

[1] $$\begin{array}{c} f_{i}=b\left(S\right)-c\left(v_{i}\right), \end{array}$$

where S is the collective vigilance, b(S) is the benefit of predator detection, $v_{i}$ is the individual vigilance, and $c(v_{i})$ is the individual cost of vigilance. Below, we define each of these terms clearly and show how this model connects to several classes of existing models in the literature, including scanning models (1, 26, 46), probability models (9, 38, 47, 48), and hazard survival models (37, 49, 50).

Throughout our analysis, we primarily work with this model without relying on specific forms for the benefits and costs of vigilance. Analyzing vigilance in this way means that our results apply broadly, although some of the analytical treatment is necessarily abstract.

### 1.1. Defining Vigilance.

We consider individual vigilance, $v_{i}$, as an abstract measure of vigilance that could represent a range of physical realities, e.g. rate of scans for predators (1, 26), time spent scanning (9, 33, 37), the probability of detecting a predator (38, 48). The definition of individual vigilance is also constrained by Eq. **1** such that the benefit, b(S), is a function of the collective vigilance, which we define as an aggregate of the individual vigilances

[2] $$\begin{array}{c} S=\sum_{i}v_{i}. \end{array}$$

This definition aligns with existing literature that defines individual vigilance, $v_{i}$, as the rate of scanning for predators (1, 46). In these models, successful predator approaches require a time window, τ, in which no individual scans (assuming that if any individual spots a predator approach then they will warn the rest of the group). For each individual, the probability of detecting an approaching predator is $p=1-e^{-v_{i}\tau }$. For a collective, the probability of any (one or more) individuals detecting an approaching predator is $p_{C}=1-e^{-S\tau }$, where the collective vigilance, S, is the sum of individual vigilances. If individual scans are independent Poisson processes with rate $v_{i}$, then the collective rate of scanning is described by a combined Poisson process with rate $S=\sum_{i}v_{i}$.

In other literature, vigilance is defined more directly in terms of the probability of an individual detecting an approaching predator, p, so that a predator approach is successful if all of the individuals fail to detect the approach in time, given by $p_{C}=1-{\left(1-p\right)}^{n}$ (9, 38, 47, 48). At first glance this seems to be incompatible with our definition in Eq. **2**. However, if we define the vigilance as $v_{i}=-ln\left(1-p_{i}\right)$, then the probability of the group detecting an approaching predator is a function of the collective vigilance, $p_{C}=1-e^{-S}$ (and the probability for a single individual is $p_{i}=1-e^{-v_{i}}$), similar to the scanning model. Note that defining vigilance in this way changes nothing about the actual system we are studying, but it does unite models that describe vigilance in terms of rates of scans for predators with those which more directly work with probabilities.

Some models in the literature are multiplicative. For example, hazard models assume that fitness benefits accrue until an individual is predated (37, 49, 50). In a simple form, expected fitness can be written as $f_{i}=T\rho_{i}$, where T is the expected survival time and $\rho_{i}$ is the individual rate of fitness gain. To accommodate such multiplicative models in our framework, one could log-transform these models to separate them into the additive form in Eq. **1**, i.e. $\text{log}\left(f_{i}\right)=\;\text{log}\left(T\right)+\text{log}\left(\rho_{i}\right)$, so that the benefit is b(S)=log(T) and the cost is $c\left(v_{i}\right)=-\text{log}\left(\rho_{i}\right)$. The rest of our analysis then follows, since maximizing the log-transformed fitness is equivalent to maximizing the nontransformed function.

The minimal model that we present in Eqs. **1** and **2** is homogeneous in that every individual is the same in every way, although they can have different individual vigilances, $v_{i}$. We extend the minimal model toward heterogeneity in *Extensions* in the form of edge effects and energetic state. Our analysis also involves a mean-field assumption in that individuals experience the benefit of collective vigilance through an aggregate of their individual-level vigilances, S. This mean-field assumption is an approximation; in *Discussion* we explore connections to alternative theoretical approaches including private vigilance benefits and spatial models.

### 1.2. The Benefit of Collective Vigilance.

Without defining a specific form for the benefit function, b(S), we can nevertheless say something about its shape.

The benefit of collective vigilance is in reducing the threat of predation. We define the predation threat, r, as an extrinsic threat level (equivalent to the expected individual fitness cost of predation if the group was not vigilant at all). Fundamentally, the most one can do is to completely remove this threat, i.e. reduce the probability of predation to zero (or a minimum), in which case the benefit of collective vigilance would be b(S)=r. Formally, we can say that 0≤b(S)≤r. The benefit increases with collective vigilance, $\frac{\mathrm{db}}{\mathrm{dS}}>0$. However, because the maximum benefit is capped, benefits cannot increase indefinitely and they must eventually saturate. We capture this by assuming that marginal benefits are decreasing, $\frac{d^{2}b}{dS^{2}}<0$, so that the benefit function is increasing and concave.

The predation threat appears in existing literature (29, 42, 45). In our definition, the single variable r captures a range of considerations including the rate of predator approaches (37, 45), the chance of individual predation given a successful approach (37, 45), as well as the relative fitness cost of being predated (e.g. the predation threat can be expressed as a multiplication of these considerations). The level of predation threat is also influenced by other group antipredator benefits such as dilution (3, 24, 46) and confusion (3, 6, 51) effects, which we assume are fixed for a given group size.

### 1.3. The Costs of Vigilance and Environmental Structure.

As with benefits, we can say something about the shape of the cost of vigilance function, $c(v_{i})$, without defining a specific form.

Individuals pay a fitness cost for maintaining vigilance. In the literature this cost is often connected (explicitly or implicitly) to the opportunity costs of foraging (27, 29, 30, 37, 42–44). Time spent looking out for predators is time not spent foraging for food (or engaging in other fitness-enhancing activities). Note that in our model the costs of vigilance do not include predation costs, which are fully captured by the benefit function as described above.

We assume that each individual can have a range of levels of vigilance, $v_{i}\in \left[0,v_{\max}\right]$, where $v_{\max}$ corresponds to the vigilance where an individual is fully committed to detecting predator approaches. We assume that $v_{\max}$ is finite, reflecting an assumption that the probability of detecting a predator can approach but never reach 1 ($p_{i}=1-e^{-v_{i}}$). Individual vigilance costs scale with the level of vigilance as described by a function $c(v_{i})$. We assume that the cost function is increasing, $\frac{\mathrm{dc}}{dv_{i}}>0$, i.e. higher levels of vigilance incur higher costs. As fitness is relative, we arbitrarily set c(0)=0.

Some habitats are approximately uniform in the sense that predator approaches can be observed equally well from any position (22) (such as an open field). In this case, we can reasonably approximate the costs of vigilance as the opportunity cost of the time not spent foraging for food (19). Following the literature, we consider that fitness costs for a low level of vigilance are relatively small (2, 37, 52), as the time spent being vigilant has a small effect on foraging efforts. How do these costs scale when an individual spends more time being vigilant? At one extreme, if an individual spent all their time being vigilant and no time foraging then they would not survive, in which case their fitness cost would be very high. We therefore assume that the costs of vigilance in uniform habitats start off shallow at low vigilance before becoming steeper at high vigilance, $\frac{d^{2}c}{dv_{i}^{2}}>0$, i.e. costs of vigilance are steepening (convex).

Other habitats have areas that have low visibility for detecting predators and other areas that provide better vantage points (such as mounds or trees) (13, 14, 16, 19, 20, 22, 29, 30). The cost of vigilance in this case starts off steep, as in order to increase vigilance the individual must remove themselves from foraging and move to the vantage point. However, once at the vantage point the cost of increasing vigilance is relatively cheap: Once you are up the tree you may as well keep your eyes peeled. These environments are characterized by flattening (concave) costs of vigilance $\frac{d^{2}c}{dv_{i}^{2}}<0$.

Of course, vigilance costs can be more complicated and can have regions of convexity and concavity. We discuss these considerations in *Extensions*.

## 2. Results

### 2.1. Selfish Groups.

Selfish groups are made up of N individuals who are incentivized to maximize their own individual fitness (37), where fitness is relative to a reference population larger than the group (Eq. **1**). Because the benefit of vigilance depends only on the level of collective vigilance, $S=\sum v_{i}$, all group members share the same benefit, b(S), as well as the same marginal benefit $\frac{\mathrm{db}}{\mathrm{dS}}$. Following existing work (34), we model behavior as a dynamic game whereby individuals adapt their behavior to the ecological context and the vigilance levels of the rest of the group.

Consider first an environment without vantage points. Individuals increase their vigilance at the opportunity cost of foraging, with steepening (convex) vigilance costs such that marginal costs increase with the individual level of vigilance. If Alice has higher vigilance than Bob, then she is paying a higher marginal cost but receiving the same benefit, so Bob is more incentivized than Alice to increase his vigilance. If the collective vigilance is high enough then Alice is incentivized to reduce her vigilance while Bob increases his. Their vigilances converge. Extending this argument to the entire group, the only stable outcome is for everyone to have the same vigilance, i.e. a many-eyes strategy.

Now consider an environment with a vantage point, such as a tree or mound. Reaching the vantage point is costly, but once there increasing vigilance is relatively cheap, so that costs are flattening (concave). If the predation threat is high enough then Alice finds it beneficial to pay the cost of climbing the vantage point to keep watch. Once there, Alice has low marginal costs while Bob (still on the ground) has high marginal costs. As they share the same marginal benefit, Alice is incentivized to increase her vigilance while Bob reduces his to zero. This divergence generates a sentinel style strategy with one or a few individuals maintaining high vigilance while the rest of the group have zero vigilance.

More formally, each individual’s incentive to change vigilance is determined by the slope of their fitness function

[3] $$\begin{array}{c} \frac{df_{i}}{dv_{i}}=\frac{db(S)}{dv_{i}}-\frac{dc(v_{i})}{dv_{i}}. \end{array}$$

Since $\frac{\mathrm{db}}{dv_{i}}=\frac{\mathrm{db}}{\mathrm{dS}}$ is the same for all group members, individuals differ only in their marginal costs. When costs are steepening (convex), individuals with lower vigilance have lower marginal costs and are more incentivized to increase their vigilance, driving vigilances together. When costs are flattening (concave), the individual with higher vigilance has the lower marginal cost, driving vigilances apart.

**Fig. 1.**
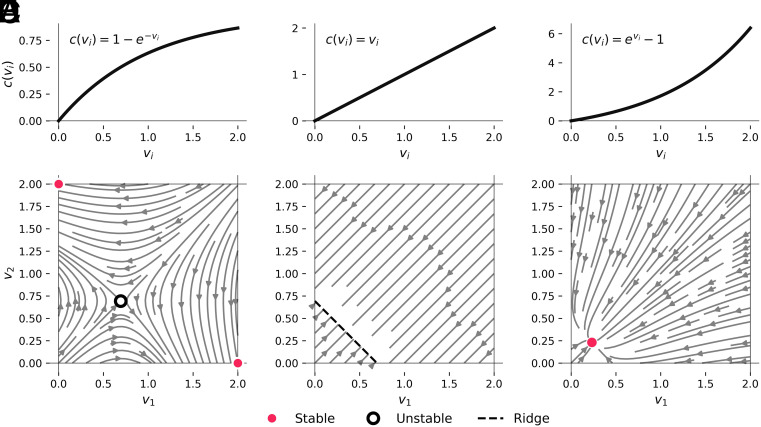
Selfish groups with (*A*) flattening (concave) vigilance costs, (*B*) linear costs, and (*C*) steepening (convex) costs. Corresponding phase portraits (*D, E, F*) show strategy dynamics with a group of size N=2, with individual vigilances of $v_{1}$ (x-axis) and $v_{2}$ (y-axis). (*A*) With flattening (concave) costs there is (*D*) a single dynamically unstable interior equilibrium and N symmetric stable equilibria on the edges of the strategy space. (*B*) With linear vigilance costs there is (*E*) an N−1 dimensional ridge of neutrally stable equilibria with a fixed optimal collective vigilance. (*C*) With steepening (convex) costs there is (*F*) a single interior many-eyes stable point with all N watchers having the same individual vigilance.

We generalize this argument with a dynamical systems analysis (53). Assuming that individuals adjust their vigilance in response to others, the system evolves according to the fitness gradients. Fig. 1 shows phase portraits for a group of N=2, with the flow lines showing how the group strategy is expected to change as individuals move toward higher fitness. The form of stable strategies is determined by the eigenvalues of the Jacobian matrix (see *SI Appendix* for full analysis), which in turn depend on the shape of the vigilance cost function:

- Flattening (concave) costs (Fig. 1 *A* and *D*): All interior points are unstable so that only edge (sentinel) solutions are stable. Individuals diverge to either zero or maximum vigilance, with at most one individual at an intermediate vigilance.
- Linear costs (Fig. 1 *B* and *E*): Interior solutions form a neutrally stable ridge. Strategies converge toward the optimal collective vigilance $S^{*}$, with any distribution of vigilances being stable.
- Steepening (convex) costs (Fig. 1 *C* and *F*): Interior (many-eyes) solutions are stable. All individuals converge to the same level of vigilance.

For completeness we investigated other solution concepts (*SI Appendix*). A Nash equilibria analysis broadly agrees with the dynamical systems analysis. For convex (steepening) costs only many-eyes strategies are Nash equilibria. For sufficiently concave (flattening) costs, only sentinel style strategies (with vigilance concentrated in a few individuals) are Nash equilibria. However, if costs are concave but benefits are more concave, there exist both many-eyes and sentinel Nash equilibria. An evolutionarily stable strategy (ESS) analysis finds that only many-eyes strategies are evolutionarily stable; sentinel style strategies are not stable since mutants with low vigilance can always free-ride and invade these populations. These concepts address different biological questions: Nash equilibria describe static best responses, and ESS analysis is suitable when investigating how genetic traits invade populations (or resist invasion) (54, 55). Empirically, vigilance behavior is flexible as individuals adjust their vigilance in response to group composition and the presence of sentinels (5, 16, 18, 56). For this reason dynamical systems analysis, which captures ongoing behavioral adjustments, provides the most appropriate approach.

### 2.2. Cooperative Groups.

Cooperative groups are made up of N individuals who are each aiming to maximize the average expected fitness of the group members (37). The optimal strategy is to distribute vigilance in the most efficient way.

When costs are steepening (convex), it is most efficient to exploit the low marginal costs at low vigilance by distributing vigilance throughout the group in a many-eyes strategy: If Alice has higher vigilance than Bob then the average cost would be lower if Alice reduced her vigilance slightly and Bob increased his by the same amount. When costs are flattening (concave; environments with a vantage point), those with high vigilance have lower marginal costs so it is more efficient to concentrate vigilance in as few individuals as possible.

Formally, the average fitness is given by

[4] $$\begin{array}{c} \overset{¯}{f}=b(S)-\overset{¯}{c(v_{i})}, \end{array}$$

where $\overset{¯}{f}=\frac{\sum_{i}f_{i}}{N}$ and $\overset{¯}{c(v_{i})}=\frac{\sum_{i}c\left(v_{i}\right)}{N}$. For any fixed collective vigilance, $S=S^{*}$, the benefit $b(S^{*})$ is fixed. In this case the distribution of vigilance $v=[v_{1},v_{2},...,v_{N}]$ that maximizes the average fitness is that which minimizes the average costs,

[5] $$\begin{array}{c} \max_{v}\overset{¯}{f}=\min_{v}\overset{¯}{c},\;S=S^{*} \end{array}$$

**Fig. 2.**
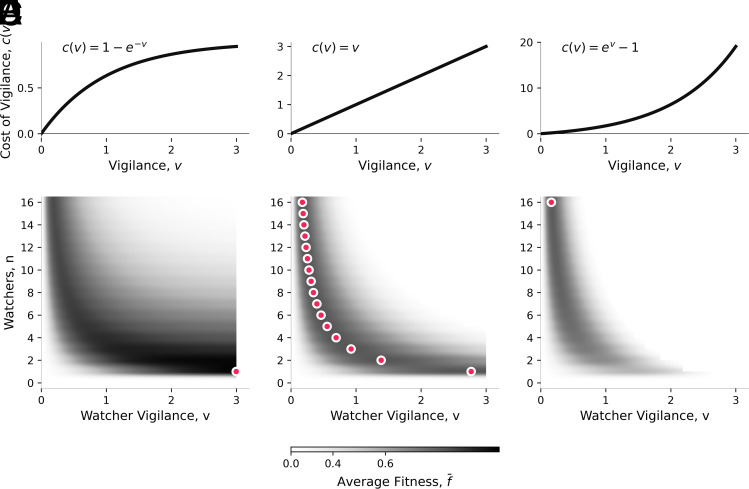
Cooperative groups with N=16 individuals. The vigilance cost functions (*A*–*C*) determine the fitness landscapes (*D*–*F*) over the number of watchers, n, with nonzero vigilance, and the vigilance of these watchers, v. Optimal strategies are denoted with dots. (*A*) Flattening (concave) vigilance costs favor (*D*) a sentinel style strategy with a single watcher, N=1, with high vigilance. (*B*) Linear vigilance cost functions lead to (*E*) indifference between strategies along an optimal ridge. (*C*) Steepening (convex) vigilance costs favor (*F*) a many-eyes strategy with the entire group, N=16, maintaining a low individual vigilance. Heatmaps only show positive average fitness, otherwise white.
- Linear costs (Fig. 2 *B* and *E*): The group is indifferent because all distributions of vigilance for a given level of collective vigilance, $S^{*}$, have the same average cost.
- Steepening (convex) costs (Fig. 2 *C* and *F*): Distributing vigilance equally across the many-eyes of the group minimizes average cost and maximizes fitness.

The optimal strategy is determined by the curvature of the vigilance cost function:

- Flattening (concave) costs (Fig. 2 *A* and *D*): Concentrating vigilance in one or a few individuals minimizes costs. If there is no maximum vigilance then a single individual with high vigilance (and others with no vigilance) is optimal, otherwise multiple high vigilance individuals can be preferred.

If vigilance is concentrated in a single individual then the average cost of vigilance across the group is $\frac{c(S^{*})}{N}$. If the same collective vigilance is spread equally through the many-eyes of the group, then the average cost of vigilance is $c(\frac{S^{*}}{N})$. Comparing these two averages, we can see (by Jensen’s Inequality) that which is greater depends on the convexity/concavity of the costs of vigilance: when vigilance costs are steepening (convex) then the many-eyes strategy has lower costs; when vigilance costs are flattening (concave) then the sentinel style strategy has lower average costs. See the SI for a general proof.

Crucially, this result holds for any level of collective vigilance: The question of how to distribute vigilance (a many-eyes or sentinel style strategy) is separate from the question of the optimal level of collective vigilance.

### 2.3. Simulations.

**Fig. 3.**
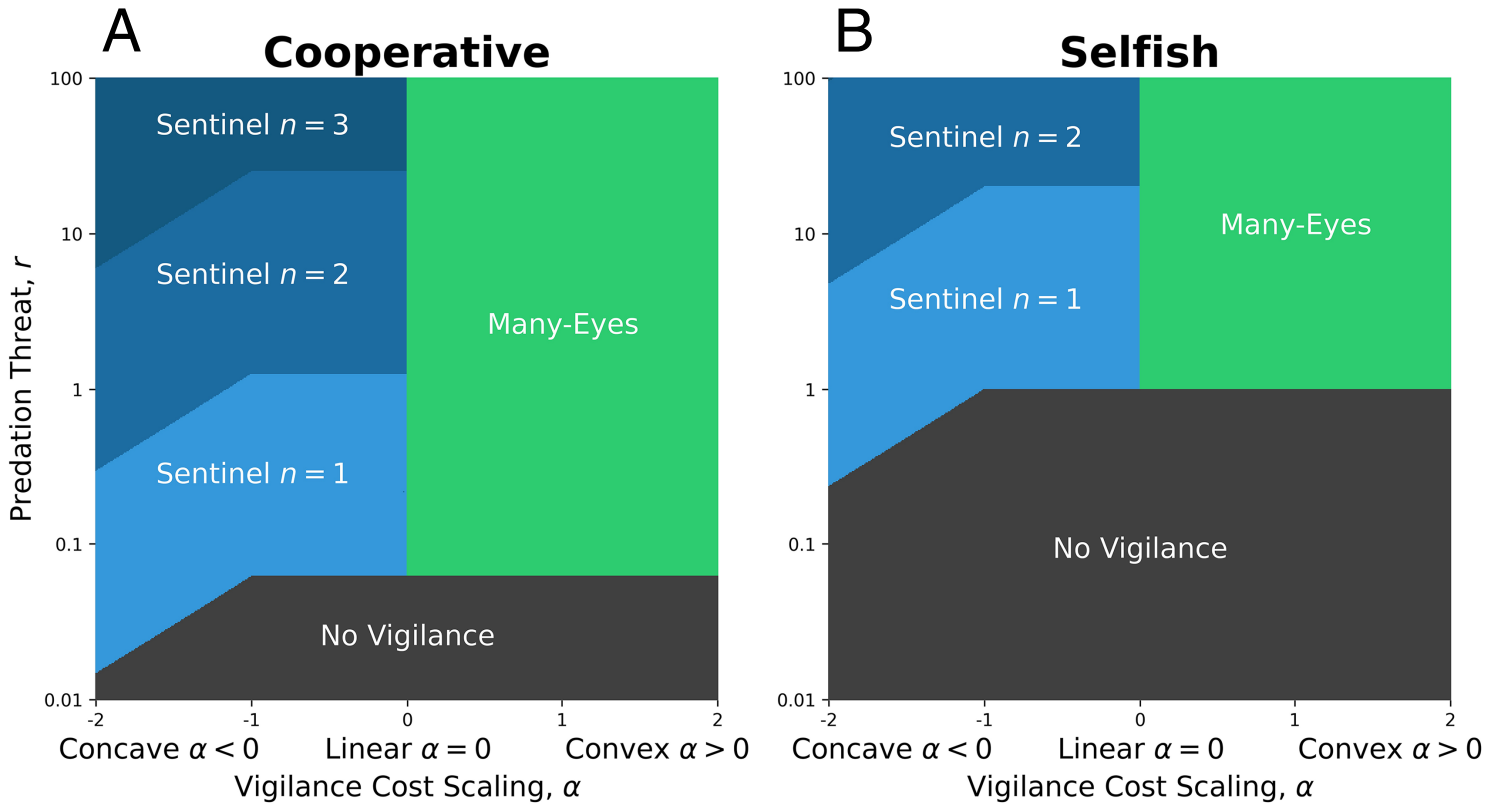
Simulated vigilance behavior in (*A*) cooperative and (*B*) selfish groups with varying scaling in vigilance costs (x-axis) and predation threat levels (y-axis log scale). In both cooperative and selfish groups, there is a clear dichotomy where we see distributed vigilance (many-eyes, green) with steepening (convex) costs and concentrated vigilance (sentinel, blue) with flattening (concave) costs.

We verify the analysis through simulations with varying social context (selfish or cooperative), shapes of vigilance cost curves, and predation threat level, r (Fig. 3).

For each of the selfish and cooperative contexts, we ran a simulation for each point in a 500 × 500 parameter space described by the convexity/concavity, α∈[−2,2] (concave for α<0 and convex for α>0) and predation threat level r∈[0.01,100]. Each simulation was initialized with a group of N=16 individuals, each with a uniformly randomly generated vigilance $v_{i}\in \left[0,v_{\max}=10\right]$. At each timestep, an individual within the group was randomly selected to modify their vigilance to selfishly maximize their own fitness (Fig. 3*A*) or to cooperatively maximize the average group fitness (Fig. 3*B*), given the rest of the group’s strategy. New vigilances were found using a scalar minimization algorithm over the interval $v_{i}\in \left[0,v_{\max}=10\right]$ [using the Python package scipy (57)]. Each simulation was run until the group vigilances converged. Note that we set a maximum vigilance, $v_{\max}$, which leads to strategies with more than one sentinel as predation threat increases. Note that these simulations use a specific functional form for the benefit and cost functions (*Materials and Methods*); this form was chosen for demonstration purposes and the earlier analytical results do not depend on this specific function. We chose a group size of N=16, representative of typical group sizes in which both forms of collective vigilance are studied empirically [e.g. sentinels in groups of 2 to 30 (13, 16, 18, 21, 36), many-eyes in groups of 3 to 80 (8, 10, 12) and much larger (5, 40)]. Our analytical results are independent of group size, which we demonstrate in *SI Appendix* by replicating these simulations with N=4 and N=64.

The simulations match the predictions from the analysis: Many-eyes strategies appear when vigilance costs are steepening (convex) and sentinel style strategies when vigilance costs are flattening (concave). Throughout the parameter space the cooperative groups have higher (or equal) average fitness than selfish groups.

### 2.4. Extensions.

Our analysis so far has been restricted to groups made up of identical individuals with relatively simple cost functions. Here, we discuss how the model can be extended toward more realistic scenarios.

**Fig. 4.**
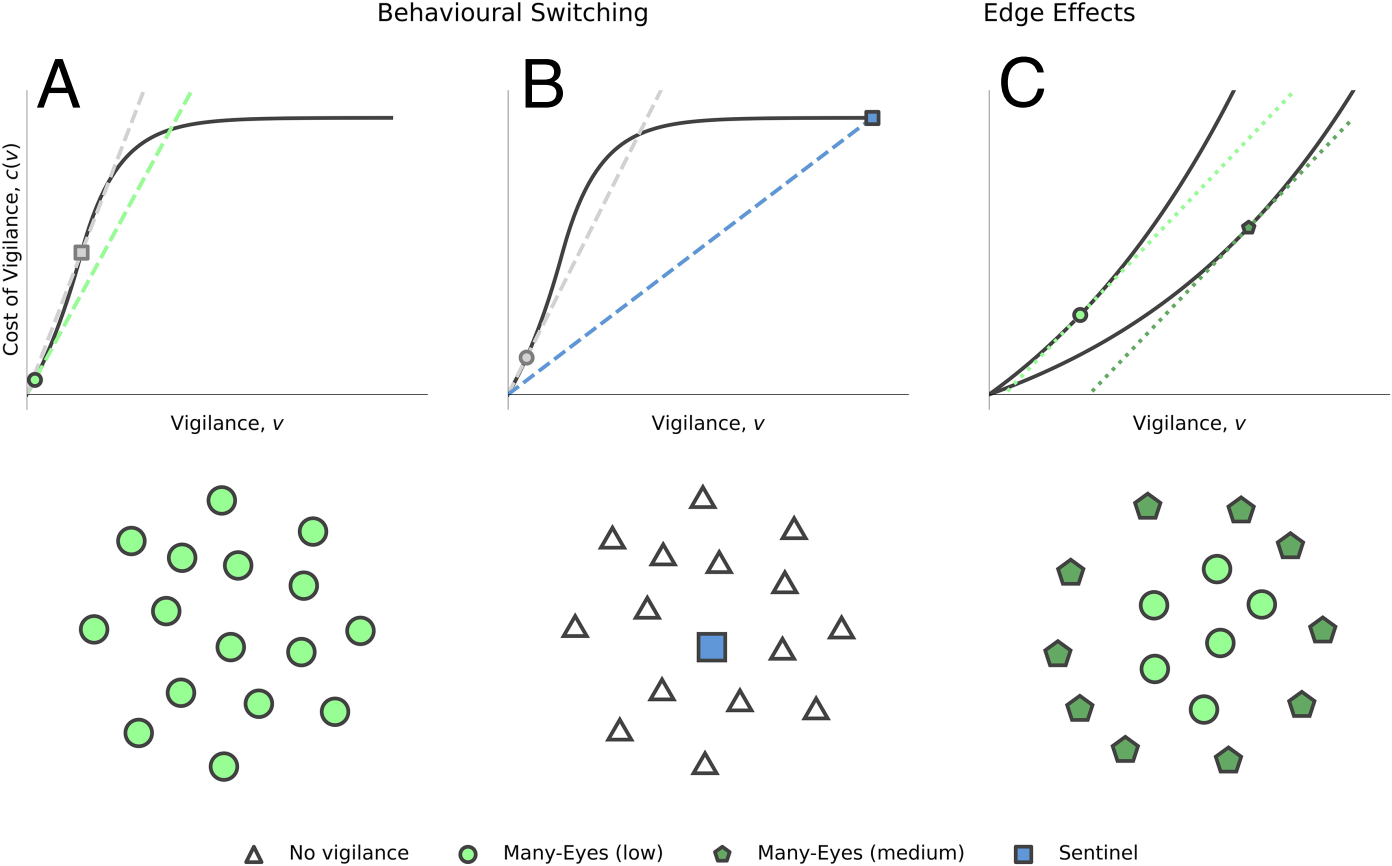
Behavioral switching and edge effects. (*A* and *B*) Example of behavioral switching with S-shaped cost curves. (*A*) At low predation threat, the many-eyes strategy (green circles) has a lower average vigilance cost (gradient of green dashed line) than a sentinel-style strategy with concentrated vigilance (gradient of dashed gray line). (*B*) At high predation threat levels, a sentinel-style strategy (blue square) has a lower average vigilance cost (gradient of dashed blue line) than a many-eyes strategy (gradient of dashed gray line). (*C*) Example of edge effects with heterogeneous cost curves. Lower costs at the edge of the group leads to higher vigilance at the edge (dark green pentagons) than the interior (green circles), with all individuals having the same marginal costs (dotted tangents).

While environments with vantage points have flattening (concave) regions at high vigilance, it is reasonable to also expect a region of steepening (convex) costs at low vigilance (those foraging on the ground have the option to keep a low-level lookout, even when visibility is low), forming a sigmoidal (S-shaped) vigilance cost function. These functions can support either many-eyes (distributed vigilance) or sentinel-style (concentrated vigilance) behavior, with each being locally optimal in the convex and concave regions respectively. Which is globally optimal depends on the full environmental conditions. In Fig. 4 we show an example of a sigmoidal cost function. We simulated the optimal strategy for cooperative groups in the same way as described in *Simulations*. At low predation risk, the optimal level of collective vigilance is relatively low, so that it is more efficient to operate in the convex region of the curve, spreading the vigilance in a many-eyes strategy to exploit the low marginal costs at low vigilance (Fig. 4*A*). At high predation risk the optimal collective vigilance is greater, such that a many-eyes strategy now becomes less efficient and the group finds it worthwhile to switch to concentrate vigilance in a sentinel-style strategy, now in the concave region of the vigilance cost curve (Fig. 4*B*). The example given is for cooperative groups; we also simulated behavioral switching in selfish groups (which occurs at a higher predation threat rate). This simulated behavioral switching qualitatively matches empirical associations between the prevalence of sentinels and levels of predation threat (22, 36, 58), and behavioral switching in meerkats such that the vigilance of foragers drops when sentinels are in position (16).

Real groups have heterogeneity due to spatial positions (8, 45), energetic state (16, 59), genetic variation (30), and mixed species groups (43, 60). In cooperative groups, heterogeneity in benefits will have no qualitative effect, as the group will still optimize the averaged benefit function. In selfish groups, heterogeneity in benefits is mathematically equivalent to heterogeneity in costs (as adaptive costs are relative to benefits).

With heterogeneous steepening (convex) vigilance costs, our model predicts many-eyes strategies with both cooperative and selfish groups, with differential levels of vigilance such that all individuals share the same marginal cost. Those with lower relative costs (e.g. peripheral individuals facing higher predation risk) maintain higher vigilance (Fig. 4*C*). These kinds of edge effects have been observed empirically in starlings (41) and brent geese (40).

With heterogeneous flattening (concave) vigilance costs, we still see sentinel style concentrated vigilance strategies. For cooperative groups, the optimal behavior is for vigilance to be concentrated in the individual with the lowest cost of vigilance. For selfish groups, there can be multiple equilibrium solutions with some individuals who would stably play the high vigilance role, but others who would not. For example, those who are satiated may find it beneficial to act as sentinel (16).

**Fig. 5.**
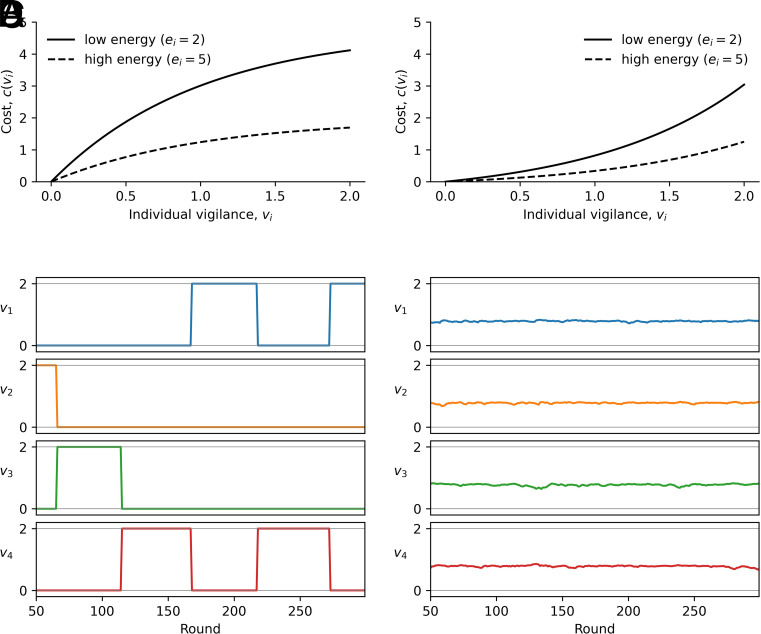
Energy-dependent simulations with stochastic foraging in selfish groups with (*A*) flattening (concave) costs and (*B*) steepening (convex) costs. Vigilance costs are inversely scaled by energy state. (*A*) Flattening (concave) costs with energetic scaling generate (*C*) turn-taking of a sentinel role with maximum vigilance while others have zero vigilance. (*B*) Steepening (convex) costs with energetic scaling generates (*D*) no distinct roles with all individuals maintaining a similar level of vigilance with relatively small fluctuations.

A key feature of sentinel behavior is turn-taking (16, 22, 34, 36), which is not addressed by our static analysis. We introduce a minimal extension with dynamic energy states, similar to existing state-dependent models (29, 34). Energy is depleted through a constant metabolic expenditure and replenished through stochastic foraging returns that depend on each individual’s vigilance level. Vigilance costs are scaled inversely with energetic state, so that satiated individuals face lower opportunity costs of vigilance (Fig. 5 *A* and *B*). Flattening (concave) costs generate turn-taking (Fig. 5*C*): A sentinel maintains high vigilance while satiated until its energy depletes, at which point a higher-energy individual takes over. With steepening (convex) costs the same energy dynamics produce only small fluctuations around a symmetric many-eyes equilibrium (Fig. 5*D*). Turn-taking thus emerges only with flattening (concave) costs, without any explicit coordination mechanism; this is consistent with state-dependent models and empirical observations that sentinel duty in meerkats is associated with satiation (16, 29, 34). Results shown are for selfish groups; cooperative groups show the same pattern of turn-taking with concave costs but not convex costs (*SI Appendix*). We also find this qualitative pattern under an alternative mechanism whereby energetic state sets the order in which individuals update their vigilance, rather than scaling the cost (*SI Appendix*).

## 3. Discussion

Previous theoretical work on collective vigilance focuses on specific species, behaviors, or social contexts (1, 26, 29, 34, 37), leaving what Bednekoff (22) calls “large gaps in knowledge” in the ecological conditions that lead to sentinel or many-eyes behavior. In order to address this gap we derive the behavior that maximizes fitness under varying ecological conditions. We find that the curvature of individual vigilance costs determines whether the equilibrium vigilance profile is distributed across the many-eyes of the group or else concentrated in one (or a few) individuals taking on sentinel roles while others have no vigilance. Specifically, we find that concentrated vigilance (sentinels) is favored when there is an exploitable vantage point such that the costs of increasing vigilance are flattening (concave) and distributed vigilance (many-eyes) when there is no vantage point and costs are steepening (convex). Notably, this same dichotomy holds in both selfish and cooperative groups. Our approach is grounded in the optimality tradition in behavioral ecology, and ultimately rests on the assumption that adaptive pressures favor expressed behavior that increases fitness (61–63). Precisely, we assume that selection acts on context-dependent behavioral strategies (rather than genetically fixed vigilance levels), matching empirical evidence that individuals adjust their vigilance depending on the predation threat and vigilance of others (5, 16, 18, 45).

Our model predicts that sentinel behavior will only emerge in ecological contexts where at least part of the cost function is flattening (concave), i.e. a behavioral option with steep initial costs followed by shallower marginal costs. For example, climbing a tree is costly but once up there it costs little extra to maintain a dedicated lookout. This matches the empirical pattern of sentinels occurring in species that forage on the ground in an area of low visibility for predator approaches but that have the availability of vantage points away from the ground with much better visibility (13, 14, 16, 19, 20, 22, 29, 30). However, the availability of a vantage point does not directly imply sentinel behavior, and species must also have adaptations to be able to exploit that vantage point (e.g. perching in babblers, bipedal stances in meerkats, behavioral adaptations to coordinate vigilance). In the context of our model, one can say that vigilance costs depend on both the environment and species’ traits. For example, many species of babblers (the genus *Turdoides*) live in habitats with vantage points and exhibit sentinel behavior (13, 14, 58, 59) even in habitats where other birds do not (22). We expect this pattern to hold more generally, and we predict that cross-species analyses of sentinel behavior will reveal a strong association with the availability of vantage points in evolutionary habitats as well as clustering within closely related species, which could be addressed through a meta-analysis of existing empirical data.

We extend our model to more realistic sigmoidal vigilance costs, which we show can generate behavioral switching from distributed (many-eyes) to concentrated (sentinel) strategies as predation threat rises. This theoretical finding aligns with empirical observations that sentinel behavior occurs more often when predation threats are high (22) as observed in Florida scrub jays (36) and pied babblers (58); as well as the discrete behavioral switching between many-eyes and sentinel strategies seen in meerkats (16). There is evidence that pied babblers adapt their foraging behavior in the presence of sentinels, including far fewer scans for predators (64), but this does not appear to be universal with an observational study in Arabian babblers finding no clear change in foraging behavior in the presence of sentinels (65). Our work predicts that behavioral switching should be quite common in sentinel species, and we encourage further empirical work across a wider range of species to i) test whether groups switch from many-eyes to sentinel behavior with increased predation threat and ii) test whether forager vigilance drops when a sentinel is on guard (both could be tested through either naturalistic observations or simulated interventions).

We also extend our model to show how heterogeneity in the costs (and benefits) of vigilance can generate empirically observed edge effects where individuals on the edge of a group are more vigilant than those in the interior (5, 40, 41). Finally, we show that adding energetic states to the model generates turn-taking of high vigilance roles in the case of flattening (concave) costs; in the case of steepening (convex) costs we do not see turn-taking but instead fluctuating distributed vigilance. There are many other realistic assumptions that could be incorporated, such as forager density influencing vigilance efficiency (66, 67), variable feeding rates (37, 66), and dilution and confusion effects (68).

Our analysis assumes that the individual benefit of collective vigilance can be expressed as a function of the sum of individual vigilances (a mean-field approximation). This assumption encompasses a large set of potential models but not all: It does not include more complex scanning models (26, 66, 67), safer sentinel positions (16, 29, 34, 59), or spatial structure (66, 67). The strength of our minimal approach is that it generates a wide range of behaviors within a single framework (many-eyes and sentinels in both selfish and cooperative groups) and identifies the ecological conditions that determine which behavior should appear. The associated limitation is that we do not include assumptions that may in fact be important components of real-world vigilance behavior.

The mean-field approximation assumes perfect information sharing of predator threats. In reality, first observers can be more likely to escape a predator approach, creating private benefits of vigilance which are a key component of some modeling approaches (29, 34, 37, 45, 69, 70). These private benefits may also extend to neighbors where predator alarms spread through a network, as addressed in spatially explicit models (69, 71). In these cases there is still a collective action problem with social and private components, which can be incorporated in our analysis such that the curvature of the net private cost function (combining private costs and private benefits) will determine whether the optimal vigilance is at an intermediate level or is driven to extremes. We therefore speculate that the many-eyes/sentinel dichotomy will still emerge locally in such systems, and we encourage further work to formalize this.

Our model is a synthesis of decades of insightful empirical and theoretical work. However, there are fundamental limitations on empirical work that may bias the behavioral patterns that we are able to observe. Collective vigilance is fundamentally difficult to measure (2, 5). Much theoretical and empirical work on many-eyes is quantified by the raising of the head to scan for predators (1, 2, 5, 26, 72), but species can have multiple vigilance behaviors including maintaining a low-level vigilance while their head is down (2, 5, 52, 73). Sentinels are associated with vantage points (13, 22), but this raises issues in that individuals on vantage points may be more likely to be observed by empiricists, it is not always clear whether an individual on a vantage point is acting as a sentinel for predators (5, 15, 22), and in any case we do not have a precise definition of a vantage point. These issues are limitations not only to our model but to the wider body of research in collective vigilance; our framework points to the importance of developing alternative empirical methods to quantify vigilance and its costs, and we suggest that a useful definition of a vantage point is the availability of a behavioral option with flattening (concave) vigilance costs. While we leave this as an open question, a step toward measuring vigilance cost curves would be quantifying foraging efficiency (i.e. food intake) as a function of vigilance level (i.e. probability of detecting an approaching predator). We also suggest that experimental manipulation of vantage points (e.g. adding or removing elevated positions) could both help quantify vigilance cost curves and test whether groups switch between many-eyes and sentinel strategies in response.

The model here is focused on animal groups. However, we hypothesize that our findings will also apply to other systems. While speculative, we note what appear to be similar many-eyes and sentinel patterns in other domains. In human society, we see social vigilance structures that resemble many-eyes [neighborhood watch (74), public vigilance campaigns on public transport (75)] and sentinel (lifeguards, security guards) behaviors. In technological systems, we see approaches with many low-cost sensors [distributed sensor networks (76), cybersecurity (77)] and singular high-cost sensors (space telescopes, radar installations). And in biological morphology, we see many-eyes style sensory arrays [compound eyes (78), touch] and singular complex sensory organs [camera eyes (78), inner ears]. While these domains have their own constraints and complexities, our model does not depend on animal groups and instead suggests a many-eyes/sentinel dichotomy in sensory systems across domains. More broadly, the dichotomy may apply to any collective action problem where individuals contribute additively to a shared benefit at individual cost (79), such as group navigation (80, 81) and collective sensing (82, 83). Our analysis suggests that the shape of the individual cost function determines whether effort is likely to be distributed or concentrated.

## 4. Materials and Methods

Our analysis does not depend on specific forms for the benefit or cost functions, beyond the assumptions laid out in *A Model of Collective Vigilance*. However, we required specific forms in order to generate explanatory figures and simulations.

Following common forms in the literature (1, 26, 46), for the benefit of collective vigilance we used

[6] $$\begin{array}{c} b\left(S\right)=r\left(1-e^{-S}\right). \end{array}$$

This meets our conditions of increasing but saturating benefits of collective vigilance, and zero benefit at zero collective vigilance.

For vigilance cost functions we used the form

[7] $$\begin{array}{c} c\left(v\right)=\frac{e^{\alpha v}-1}{\alpha }. \end{array}$$

This meets our conditions of an increasing cost function, zero cost at zero vigilance, and parameterizes the convexity/concavity of the cost function with α<0 being concave and α>0 being convex. We chose this function in part because it has a similar derivative to the benefit function, so that the interior equilibrium of the fitness function is solvable analytically.

The cost function in Eq. **7** reduces to linear costs as α→0. To show this, we first note that the Taylor series of $e^{x}$ around x=0 is $e^{x}=\sum_{k=0}\frac{x^{k}}{k!}$. We can write

[8] $$\begin{array}{c} \lim_{\alpha \to 0}c\left(v\right)=\frac{(1+\alpha v+\frac{{\left(\alpha v\right)}^{2}}{2!}+...)-1}{\alpha }, \end{array}$$

which simplifies to

[9] $$\begin{array}{c} \lim_{\alpha \to 0}c\left(v\right)=v+\frac{\alpha v^{2}}{2!}+∼O\left(\alpha^{2}\right), \end{array}$$

which converges to $\lim_{\alpha \to 0}c\left(v\right)=v$.

We can express the cost function (Eq. **7**) in terms of the probability of an individual spotting an approaching predator, $p=1-e^{-v}$, for some values of α:

- For α=−1, concave vigilance costs, c(p)=p, i.e. linear in the probability of spotting an approaching predator.
- For α=0, linear vigilance costs, c(p)=−ln(1−p), i.e. logarithmic in the probability of spotting an approaching predator.
- For α=1, convex vigilance costs, $c\left(p\right)=\frac{p}{1-p}$, i.e. the odds ratio of spotting an approaching predator.

The boundary between many-eyes and sentinels corresponds to α=0, i.e. logarithmic costs in terms of probability c(p)=−log(1−p). This means that many-eyes strategies are favored when costs are more convex than this and sentinel style strategies when costs are more concave.

In *Defining Vigilance* we show that for multiplicative models the cost function can be written in terms of the rate of fitness gain, ρ, as c=−log(ρ). The boundary case corresponds to a rate of fitness gain that is linear in the probability of spotting a predator, ρ=1−p. This means that for multiplicative models many-eyes is preferred when the rate of fitness gain is decreasing concave in the probability of spotting a predator (it does not cost much to increase vigilance at low levels) while sentinel style strategies are preferred when the rate of fitness gain is decreasing convex in the probability of spotting a predator (it costs a lot to increase vigilance at low vigilance, but less at high vigilance).

For the energy-dependent simulations (Fig. 5), we used a group of N=4 individuals with energy states $e_{i}\in \left[0,e_{\max}=10\right]$ and vigilance $v_{i}\in \left[0,v_{\max}=2\right]$. The cost function was modified to depend on energy $c\left(v_{i},e_{i}\right)=\frac{C}{\epsilon +e_{i}}\frac{e^{\alpha v_{i}}-1}{\alpha }$, where C is a scaling constant and ϵ=0.1 prevents divergence at zero energy. Each round, individuals are drawn in random order to update their vigilance by selfish best-response given the vigilances of others. Energy then updates as $e_{i}\leftarrow e_{i}+e^{-v_{i}}\eta -m$ where $e^{-v_{i}}$ is the fraction of time spent foraging, η∼Exp(1) is a stochastic foraging return, m=0.3 is the metabolic cost per round. If energy hits a bound at 0 or $e_{\max}$ then it takes on that value. We set the predation rate r=5. The simulation was run for 500 rounds with a burn-in of 50 rounds. The scaling constant C was set to 1 for convex costs (α=1) and 10 for concave costs (α=−1). The scaling, and other model parameters, were chosen to give a clear visualization of behaviors. The qualitative behavior (turn-taking for concave costs; small fluctuations in vigilance for convex costs) is robust and does not depend strongly on parameter values (*SI Appendix*).

## Supplementary Material

Appendix 01 (PDF)

## Data Availability

Simulation data is publicly available (84).
