# Supplementary material for: Many-eyes and sentinels in selfish and cooperative groups
Source: Proc Natl Acad Sci U S A. 2026 Aug 10;123(33):e2536017123. doi: 10.1073/pnas.2536017123 (PMC13486572; doi:10.1073/pnas.2536017123)
Supplement: Supplementary file 1 [file pnas.2536017123.sapp.pdf]

Supporting Information for

Many-Eyes and Sentinels in Selfish and Cooperative Groups

C Pilgrim, A M Bate, A Sigalou, M Aellen, J Morford, E Warren, C Krupenye, D Biro and R P Mann

Charlie Pilgrim.
E-mail: c.p.pilgrim@leeds.ac.uk

This PDF file includes:

Supporting text
Figs. S1 to S4
Table S1
SI References

Supporting Information Text

Detailed Analysis for Selfish Groups

Dynamical Analysis. We assume that individuals react to the vigilance strategies of others in order to increase their own fitness, i.e. the system will move down the fitness gradient given by $\mathbf{f}' = [\frac{df_1}{dv_1}, \frac{df_2}{dv_2}, ..., \frac{df_N}{dv_N}]$. Where all individuals have zero fitness gradient there is an equilibrium point. We are interested in whether these equilibrium points are stable or unstable.

To analyze stability we follow a standard dynamical systems analysis (see (1)). One can linearize the dynamics around an equilibrium point through a Taylor expansion. This approximation captures how small perturbations develop through the Jacobian matrix, which contains the second derivatives of the fitness functions. The eigenvalues of the Jacobian determine whether perturbations grow or decay: if all eigenvalues are negative then perturbations decay and the equilibrium is stable; if any eigenvalue is positive then perturbations grow and the equilibrium is unstable.

As described in the main paper, each individual has a fitness function

$$f_i = b(S) - c(v_i)\,. \tag{1}$$

The Jacobian has entries

$$J_{ij} = \frac{d^2 f_i}{dv_i dv_j}\,. \tag{2}$$

The off-diagonal entries ($i \neq j$) are

$$\frac{d^2 f_i}{dv_i dv_j} = \frac{d^2 b(S)}{dS^2}\,. \tag{3}$$

Note that by the chain rule $\frac{d^2 b(S)}{dv_i dv_j} = \frac{d^2 b(S)}{dS^2}\frac{dS}{dv_i}\frac{dS}{dv_j}$ and $\frac{dS}{dv_i} = \frac{dS}{dv_j} = 1$, so that $\frac{d^2 b(S)}{dv_i dv_j} = \frac{d^2 b(S)}{dS^2}$.

The diagonal entries ($i = j$) of the Jacobian are

$$\frac{d^2 f_i}{dv_i^2} = \frac{d^2 b(S)}{dS^2} - \frac{d^2 c(v_i)}{dv_i^2}\,. \tag{4}$$

The Jacobian can therefore be written as

$$J = \begin{pmatrix} \alpha-\beta & \alpha & \alpha & \cdots & \alpha \\ \alpha & \alpha-\beta & \alpha & \cdots & \alpha \\ \alpha & \alpha & \alpha-\beta & \cdots & \alpha \\ \vdots & \vdots & \vdots & \ddots & \vdots \\ \alpha & \alpha & \alpha & \cdots & \alpha-\beta \end{pmatrix}, \tag{5}$$

where $\alpha = \frac{d^2 b(S)}{dS^2}$ and $\beta = \frac{d^2 c(v)}{dv^2}$. Note that at an interior equilibrium point $\frac{d^2 c(v_i)}{dv_i^2}$ is the same for all i.

We can write this compactly as

$$J = \alpha \mathbf{1}\mathbf{1}^T - \beta I\,, \tag{6}$$

where I is the identity matrix and $\mathbf{1}^T = [1, 1, ..., 1]$ and therefore $\mathbf{1}\mathbf{1}^T$ is a matrix with all entries equal to 1.

An eigenvalue, λ_k, and associated eigenvector, $\mathbf{u_k}$, is defined by the condition $J\mathbf{u_k} = \lambda_k \mathbf{u_k}$, i.e.,

$$(\alpha \mathbf{1}\mathbf{1}^T - \beta I)\mathbf{u_k} = \lambda_k \mathbf{u_k}\,. \tag{7}$$

We assume that an eigenvector is the vector $\mathbf{u_1} = \mathbf{1}$. Substituting into Equation 7,

$$\alpha \mathbf{1}\mathbf{1}^T\mathbf{1} - \beta\mathbf{1} = \lambda_k \mathbf{1}\,. \tag{8}$$

The term $\mathbf{1}^T\mathbf{1} = N$, so that we can write

$$(N\alpha - \beta)\mathbf{1} = \lambda_k \mathbf{1}\,. \tag{9}$$

This fulfills the eigenvector condition. Therefore, $\mathbf{u_1} = \mathbf{1}$ is an eigenvector with eigenvalue $\lambda_1 = N\alpha - \beta$.

The remaining eigenvectors form a basis such that all are orthogonal to $\mathbf{u_1}$. To see this, assume an eigenvector, $\mathbf{u_2}$, orthogonal to the first eigenvector, so that $\mathbf{1}^T\mathbf{u_2} = 0$ (such as $\mathbf{u_2} = [1, -1, 0, 0, ..., 0]$). Substituting this eigenvector into Equation 7,

$$\alpha \mathbf{1}\mathbf{1}^T\mathbf{u_2} - \beta\mathbf{u_2} = \lambda_k \mathbf{u_2}\,. \tag{10}$$

By the orthogonal definition $\mathbf{1}^T\mathbf{u_2} = 0$, so the first term is equal to zero and we can write

$$-\beta\mathbf{u_2} = \lambda_k \mathbf{u_2}\,. \tag{11}$$

This fulfills the eigenvector condition, so that any off-diagonal vector (orthogonal to $\mathbf{1}$) is an eigenvector with eigenvalue $\lambda_2 = -\beta$.

If we consider a basis that includes $\mathbf{u_1} = \mathbf{1}$, we will find that there are $N-1$ vectors orthogonal to $\mathbf{1}$ (with N individuals the strategy space is N-dimensional), each of which is an eigenvector with eigenvalue $\lambda = -\beta$. This gives us the eigenvalues

$$\lambda_k = \begin{cases} N\frac{d^2b}{dS^2} - \frac{d^2c}{dv^2} & k=1 \\ -\frac{d^2c}{dv^2} & k=2,3,...,N \end{cases} . \qquad [12]$$

The eigenvalues of the Jacobian determine the stability of interior equilibria. An equilibrium is stable if *all* eigenvalues are negative, $\lambda_k < 0 \quad \forall \quad k$. Conversely, an equilibrium is unstable if *any* eigenvalue is positive, $\exists k : \lambda_k > 0$.

If costs are steepening (convex), $\frac{d^2c}{dv^2} > 0$, then the off-diagonal eigenvectors are negative. In the main text, we defined the benefit function as concave because benefits saturate, $\frac{d^2b}{dS^2} < 0$, so that the first (diagonal) eigenvalue is also negative. Therefore, all eigenvalues are negative and an interior equilibrium is stable. A many-eyes strategy involves all (or many) individuals having low vigilance, $0 < v < v_{max}$, which represents an interior equilibrium. Therefore, a many-eyes strategy is stable if vigilance costs are steepening (convex).

If costs are flattening (concave), $\frac{d^2c}{dv^2} < 0$, then the off-diagonal eigenvalues are positive, so an interior equilibrium is unstable. For dynamical instability, the sign of the first (diagonal) eigenvalue is not important because the condition is for any eigenvalue to be positive, which is fulfilled by the off-diagonal eigenvalues. Any strategy with more than 1 individual with an intermediate vigilance $0 < v < v_{max}$ represents an interior solution, and will therefore be unstable if vigilance costs are flattening (concave). Therefore, stable strategies can only exist at the edges of the strategy space, with at least $N-1$ individuals having either zero vigilance or maximum vigilance. These edge solutions correspond to a sentinel strategy with one (or a few) vigilant individuals and all others having zero vigilance.

Nash Equilibria. Nash equilibria are strategy distributions where no individual is incentivized to switch strategies. An individual i has fitness

$$f_i(v_i, \mathbf{v}_{\sim i}) = b(v_i + S_{\sim i}) - c(v_i)\,, \qquad [13]$$

where $\mathbf{v}_{\sim i}$ is the strategy vector excepting player i, $S_{\sim i}$ is the collective vigilance excepting player i, b is the benefit of collective vigilance and c is the individual cost of vigilance. We assume that benefits are increasing and concave. We assume that costs are increasing and are either convex, linear, or concave.

For a Nash equilibrium, all individuals must be playing a best response to the strategy of others, i.e. they cannot improve their expected fitness by unilaterally changing their vigilance.

Symmetric Many-Eyes Nash Equilibrium. All individuals play the same $v^* \in (0, v_{max})$. A Nash equilibrium must fulfill the first-order condition:

$$\left.\frac{db(S)}{dS}\right|_{S=Nv^*} = \left.\frac{dc(v)}{dv}\right|_{v=v^*} . \qquad [14]$$

Note that $\frac{db}{dS} = \frac{db}{dv}$. This is a Nash equilibrium if the second-order condition is fulfilled that the fitness function is locally non-convex, $\frac{d^2f}{dv^2} \leq 0$,

$$\left.\frac{d^2b(S)}{dS^2}\right|_{S=Nv^*} \leq \left.\frac{d^2c(v)}{dv^2}\right|_{v=v^*} . \qquad [15]$$

Many-eyes Nash equilibria are possible when vigilance costs are more convex than benefits.

Zero Vigilance Nash Equilibrium. All individuals have zero vigilance, $v^* = 0$. This is a Nash equilibrium if there is no level of vigilance that has a greater benefit than cost

$$b(v) < c(v) \quad \forall v\,. \qquad [16]$$

In the main paper we defined the benefit and cost functions to start at zero, i.e. $b(0) = 0$ and $c(0) = 0$. The zero vigilance Nash equilibrium locally requires

$$\left.\frac{db(S)}{dS}\right|_{S=0} \leq \left.\frac{dc(v)}{dv}\right|_{v=0} . \qquad [17]$$

As benefits (and marginal benefits) are scaled by the predation threat level, r, we can expect a zero vigilance Nash equilibrium when predation threat levels are low.

Maximum Vigilance Nash Equilibrium. All individuals have maximum vigilance $v^* = v_{max}$. This is a Nash equilibrium if there is no vigilance $v \in [0, v_{max}]$ that can benefit from deviating,

$$b(Nv_{max}) - c(v_{max}) \geq b((N-1)v_{max} + v) - c(v)\,, \quad \forall v\,. \qquad [18]$$

Locally, a Nash equilibrium requires

$$\left.\frac{db(S)}{dv}\right|_{S=Nv_{max}} \geq \left.\frac{dc(v)}{dv}\right|_{v=v_{max}}. \qquad [19]$$

This condition is strong as it requires that marginal benefits at the maximum collective vigilance of the entire group are greater than the marginal costs of each individual.

Non-symmetric Interior Nash Equilibria. We define a non-symmetric interior Nash equilibrium as requiring at least two individuals with different vigilances $v_A, v_B \in (0, v_{max})$.

They must both fulfill the first-order condition at the optimal collective vigilance S^*,

$$\left.\frac{db(S)}{dv}\right|_{S=S^*} = \left.\frac{dc}{dv}\right|_{v=v_A, v=v_B}. \qquad [20]$$

The marginal benefit is the same for both individuals, as it is a function of the collective vigilance. In order for both individuals to be at equilibrium, they must have the same marginal costs. This is not possible with convex or concave vigilance cost functions. Non-symmetric Nash equilibria are possible if the vigilance cost function is linear.

If the vigilance cost function is linear, then the fitness function is concave (because benefits are concave), and so the second-order condition of a locally concave fitness function is fulfilled. One can determine the optimal collective vigilance by finding where the marginal benefit equals the linear cost gradient. Any distribution of vigilances that sum to the optimal collective vigilance is a Nash equilibrium.

Sentinel Nash Equilibria. A sentinel Nash equilibrium has n_{int} sentinels with an interior vigilance $v_{int} \in (0, v_{max})$, n_{max} sentinels with maximum vigilance and $N - n_{max} - n_{int}$ non-sentinels with zero vigilance, such that $n_{max} + n_{int} \geq 1$.

The non-sentinels are at equilibrium if they cannot improve by deviating from zero vigilance

$$b(S^* + v) \leq c(v)\,, \quad \forall v\,. \qquad [21]$$

Locally, they must have a non-positive fitness gradient

$$\left.\frac{db(S)}{dv}\right|_{S=S^*} \leq \left.\frac{dc(v)}{dv}\right|_{v=0}. \qquad [22]$$

The sentinels at maximum vigilance cannot deviate by decreasing their vigilance. Locally,

$$\left.\frac{db(S)}{dv}\right|_{S=S^*} \geq \left.\frac{dc(v)}{dv}\right|_{v=v_{max}}. \qquad [23]$$

A sentinel with an interior vigilance must locally have zero fitness function

$$\left.\frac{db(S)}{dv}\right|_{S=S^*} = \left.\frac{dc(v)}{dv}\right|_{v=v_{int}}. \qquad [24]$$

The marginal benefit is the same for all members of the group. The marginal costs must be higher at zero vigilance than maximum vigilance. This is possible if the cost function is concave or linear,

$$\frac{d^2c}{dv^2} \leq 0\,. \qquad [25]$$

Summary. The many-eyes and sentinel conditions are not equivalent, and we can describe three distinct regions:

- $\frac{d^2c}{dv^2} < \frac{d^2b}{dv^2}$. Sentinel NE are possible but many-eyes are not.
- $\frac{d^2b}{dv^2} \leq \frac{d^2c}{dv^2} \leq 0$. Both sentinel and many-eyes NE are possible.
- $0 < \frac{d^2c}{dv^2}$. Many-eyes NE are possible but sentinels are not.

Note that dynamically stable equilibria are also Nash equilibria, but Nash equilibria are not necessarily dynamically stable. The Nash equilibrium analysis is consistent with the dynamical stability analysis in that many-eyes solutions are possible when $0 \leq \frac{d^2c}{dv^2}$ and sentinel strategies are possible when $\frac{d^2c}{dv^2} \leq 0$. However, we find a region that allows both many-eyes and sentinel Nash equilibria in $\frac{d^2b}{dv^2} < \frac{d^2c}{dv^2} < 0$; this suggests that sentinel strategies in this region are dynamically unstable Nash equilibria.

Evolutionarily Stable Strategies. A common solution concept in the literature is evolutionarily stable strategies (ESS), which was an innovation led by Maynard Smith (2, 3) with the intention of applying game theory to evolutionary settings by restricting the dynamics through which populations could change strategies, to reflect the constraints of fixed genotypes within lifetimes. Evolutionarily stable strategies ask whether a mutant can invade a population with a fixed equilibrium strategy.

Within our notation, a condition for a strategy v' to be an ESS is (2)

$$f(v_m, \mathbf{v}) < f(v', \mathbf{v})\,, \tag{26}$$

where v_m is the vigilance of a mutant, v' is the vigilance of a resident and $\mathbf{v}$ is the vector of the vigilances of the group.

We begin with the fitness equation described in the main paper

$$f_i = b(S) - c(v_i)\,, \quad S = \sum_i v_i\,, \tag{27}$$

with the terms as defined in the main paper. We assume $\frac{db}{dS} > 0$, $\frac{d^2b}{dS^2} < 0$, $\frac{dc}{dv_i} > 0$.

Many-eyes ESS. A many-eyes strategy has all individuals with the same vigilance, v^*, giving a total vigilance $S = Nv^*$.

An ESS must fulfill the first-order (best-response) condition

$$\left.\frac{db(S)}{dv}\right|_{S=Nv^*} = \left.\frac{dc(v)}{dv}\right|_{v=v^*}. \tag{28}$$

Now consider a mutant with higher vigilance $v_m > v^*$. The mutant has greater fitness if the increased benefits are greater than the increased costs, which depends on how the slopes change. If $\frac{d^2b}{dv^2} < \frac{d^2c}{dv^2}$ then the mutant's extra benefits are smaller than their extra costs, and the mutant cannot invade.

The same logic applies for a mutant with lower vigilance than v^*. In this case, the mutant cannot invade if $\frac{d^2b}{dv^2} < \frac{d^2c}{dv^2}$ because benefits reduce more quickly than costs.

Hence, a many-eyes strategy is evolutionarily stable if the vigilance costs are more convex than the benefits,

$$\frac{d^2c}{dv^2} > \frac{d^2b}{dv^2}\,. \tag{29}$$

Sentinel ESS. We assume that a sentinel strategy has n_s individuals with vigilance $v_s > 0$ and all other individuals with zero vigilance, giving collective vigilance $S = v_s$.

The sentinel's fitness is

$$f_s = b(n_s v_s) - c(v_s)\,. \tag{30}$$

A non-sentinel's fitness is

$$f_i = b(n_s v_s)\,. \tag{31}$$

Consider a mutant with vigilance v_m,

$$f_m = b(n_s v_s + v_m) - c(v_m)\,. \tag{32}$$

Trivially, the mutant fitness is higher than the sentinel's fitness for $v_m < v_s$, because the mutant is receiving the full benefit of the sentinel's vigilance without matching their costs. Therefore, a mutant can invade and a sentinel strategy is not evolutionarily stable, given these assumptions.

We would therefore predict that purely genetically determined vigilance behavior in selfish groups should only generate many-eyes strategies, and we should not see sentinels in such situations. One could expand the ESS analysis to more complicated vigilance strategies such as "*act as sentinel if no one else is and I am satiated*" (4).

Detailed Analysis for Cooperative Groups

In the main article we assume that cooperative groups are incentivized to maximize the average fitness

$$\bar{f} = b(S) - \bar{c(v_i)}\,, \tag{33}$$

For a given collective vigilance, S^*, we can ask what distribution of group vigilances, $\mathbf{v} = [v_1, v_2, ..., v_N]$, maximizes fitness. The benefit is fixed at $b(S^*)$. Maximizing average fitness is therefore equivalent to minimizing the average (or total) cost

$$\max_{\mathbf{v}} \bar{f}(S^*, \mathbf{v}) = \min_{\mathbf{v}} \frac{1}{N} \sum_{\mathbf{v}} c(v_i)\,, \quad S^* = \sum_i v_i\,. \tag{34}$$

Consider a group of individuals, with two of the individuals having different vigilances v_{high} and v_{low} such that $v_{high} \geq v_{low}$. These two individuals pay a total cost of $c(v_{high}) + c(v_{low})$.

The group could achieve the same total vigilance if these two individuals instead had vigilances $v'_{high} = v_{high} + \Delta v$ and $v'_{low} = v_{low} - \Delta v$, where we label them such that $v'_{high} \geq v'_{low}$. Note that this labeling convention holds for any Δv, including cases where the individuals' relative positions switch. Crucially, this means $v_{high} > v'_{low}$.

The change in cost paid following this switch is

$$\Delta c = c(v_{high}) + c(v_{low}) - c(v'_{high}) - c(v'_{low})\,. \quad [35]$$

We can write this as

$$\Delta c = [c(v_{\text{high}}) - c(v_{\text{high}} + \Delta v)] - [c(v'_{\text{low}}) - c(v'_{\text{low}} + \Delta v)]\,. \quad [36]$$

By our construction $v_{high} > v'_{low}$. We are therefore comparing the change in costs of two equal increments in vigilance (Δv) at two different baseline levels. The sign of Δc depends on both the sign of Δv and the convexity/concavity of the vigilance cost function:

- Convex costs with diverging vigilance ($\Delta v > 0$). The cost increase at the higher baseline is greater than the cost decrease at the lower level. Therefore $\Delta c > 0$ (less efficient).
- Convex costs with converging vigilance ($\Delta v < 0$). The cost decrease at the higher baseline is greater than the cost increase at the low baseline. Therefore $\Delta c < 0$ (more efficient).
- Concave costs with diverging vigilance ($\Delta v > 0$). The cost increase at the higher baseline is smaller than the cost decrease at the lower baseline. Therefore $\Delta c < 0$ (more efficient).
- Concave costs with converging vigilance ($\Delta v < 0$). The cost decrease at the higher baseline is smaller than the cost increase at the lower baseline. Therefore $\Delta c > 0$ (less efficient).

Putting these outcomes together, we can conclude that for convex costs it is always more efficient to have more equal vigilances, so that the optimal group strategy is an equal distribution of vigilance, i.e. many-eyes. For concave costs, it is always more efficient for vigilance to diverge, so that the optimal group strategy is to have vigilances at zero or maximum vigilance, with at most one individual with an intermediate vigilance.

This argument applies for any level of collective vigilance, and so is independent of S and independent of the specific form of the predation cost rate, $b(S)$. In plain English, sentinels are preferred over many-eyes for concave cost functions, and vice-versa for convex cost functions, and which is preferred does not depend on the level of collective vigilance.

In the concave case, if vigilance is able to increase indefinitely then it is optimal to have one individual with the full collective vigilance, $v_{sentinel} = S^*$, and everyone else with zero. However, where there is a maximum possible vigilance then it can be beneficial to have multiple sentinels.

Simulations with $N = 4$ and $N = 64$

In the main paper we simulated groups of size $N = 16$ with varying social context (selfish or cooperative), cost convexity, α, and predation threat level, r. Here we present the same analysis with smaller ($N = 4$, Figure) and larger ($N = 64$) groups. We use a smaller resolution over the parameter space with 100×100 points (as opposed to 500×500 in the main paper) to avoid excessively long simulation times with large groups.

Selfish groups show identical qualitative behaviors as a function of predation threat and cost scaling, independent of group size. Larger cooperative groups have lower predation threat thresholds for both many-eyes and sentinel behaviors.

Energy Dependent Simulation with Cooperative Groups

In the Extensions section of the main paper we show an energy-dependent simulation for selfish groups. Figure S3 shows the same simulations for cooperative groups. The simulation details are the same as for the selfish groups except that individuals are aiming to optimize the average group fitness; the predation threat level is set lower to $r = 0.5$ (compared to $r = 5$ for the selfish case) in order to cleanly demonstrate turn-taking with a single sentinel, as at $r = 5$ there are multiple sentinels on duty at a time. The same qualitative patterns emerge: turn-taking with concave costs and many-eyes with convex costs.

Robustness Checks for Turn-Taking Simulations

In the main paper we present simulations of selfish groups that show emergent turn-taking in the case of concave costs and not in the case of convex costs. Here, we analyze the robustness of these simulations to varying parameters (Table S1). For these robustness checks we vary each parameter against a baseline with $N = 4$, $r = 5$, $m = 0.3$, $e_{max} = 10$ and $C = 10$. In the main paper we show a visualization with these parameters for the concave case, but with $C = 1$ for the convex case (which is included in Table S1) as this generates a clearer visualization than $C = 10$, while the qualitative behaviour is the same.

Overall, the pattern shows turn-taking sentinels when cost scaling is concave: the mean number of individuals vigilant per round is typically much lower than the size of the group, N, indicating distinct vigilant and non-vigilant roles per round. Furthermore, the standard deviation in the mean individual proportion of time being vigilant is typically low, indicating that

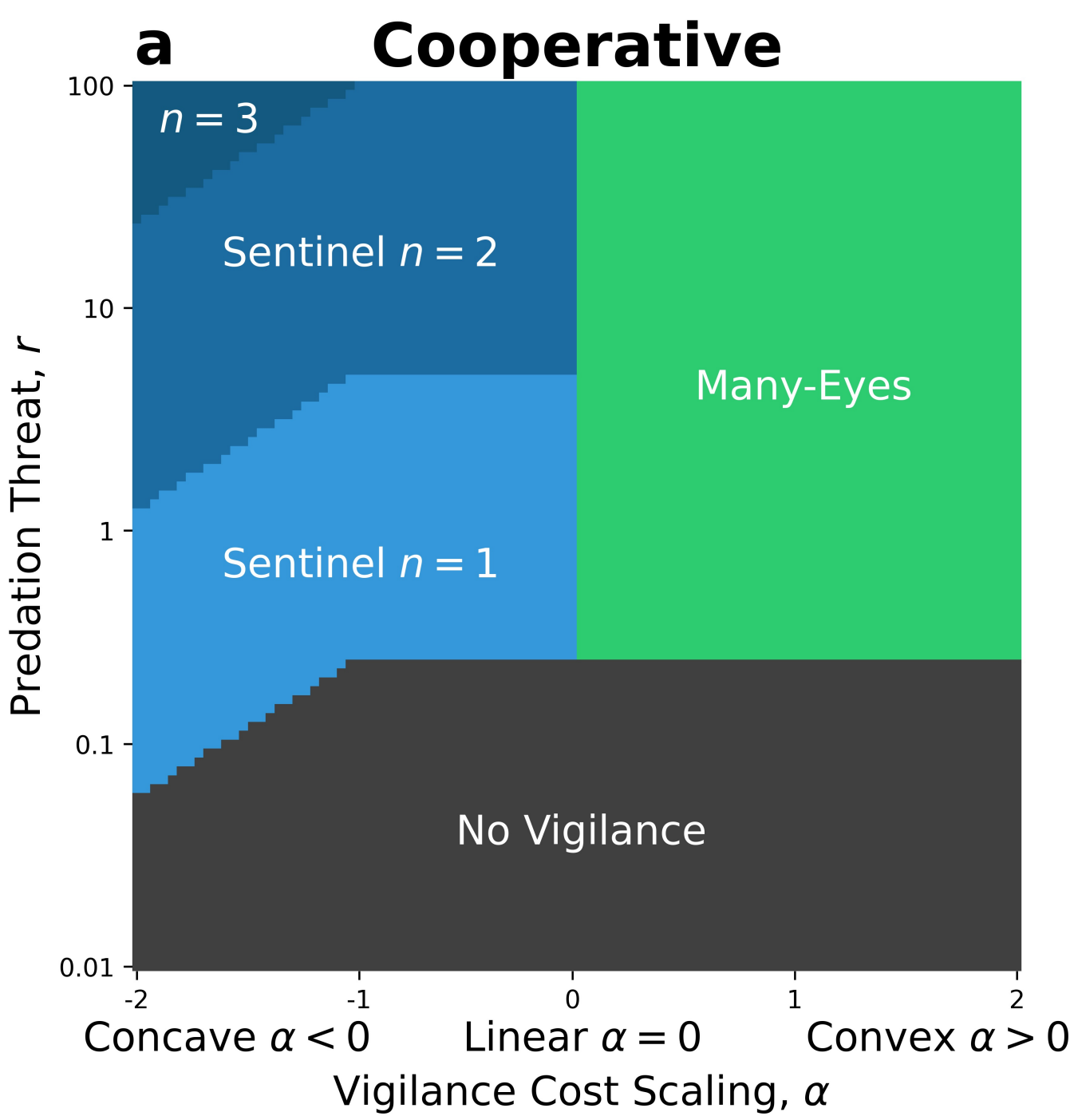

Fig. S1. Simulated vigilance behavior in groups of $N = 4$ with a) selfish and b) cooperative groups with varying scaling in vigilance costs (x-axis) and predation threat levels (y-axis log scale). In both selfish and cooperative groups, there is a clear dichotomy where we see distributed vigilance (many-eyes, green) with steepening (convex) costs and concentrated vigilance (sentinel, blue) with flattening (concave) costs.

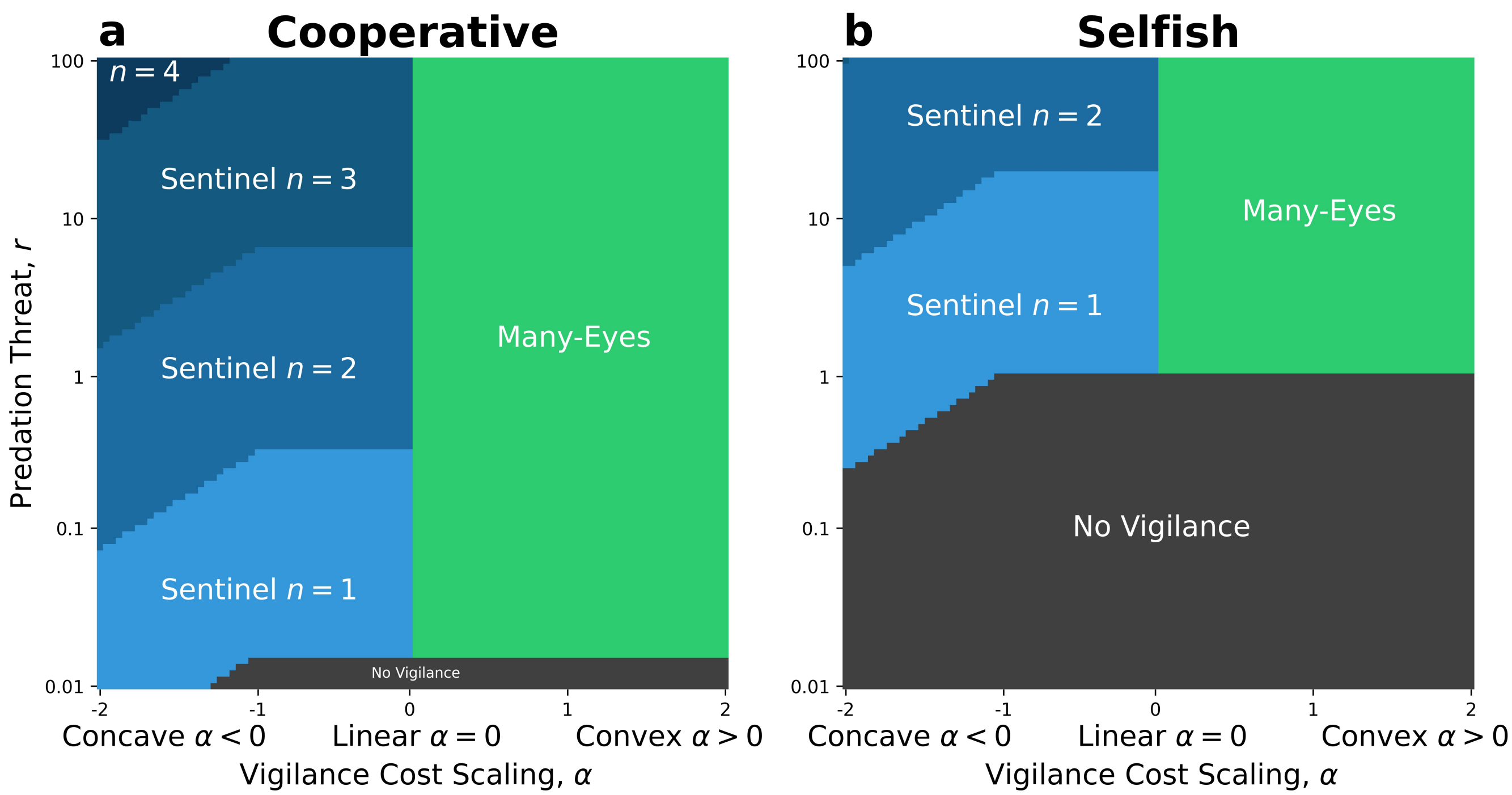

Fig. S2. Simulated vigilance behavior in groups of $N = 64$ with a) selfish and b) cooperative groups with varying scaling in vigilance costs (x-axis) and predation threat levels (y-axis log scale). In both selfish and cooperative groups, there is a clear dichotomy where we see distributed vigilance (many-eyes, green) with steepening (convex) costs and concentrated vigilance (sentinel, blue) with flattening (concave) costs.

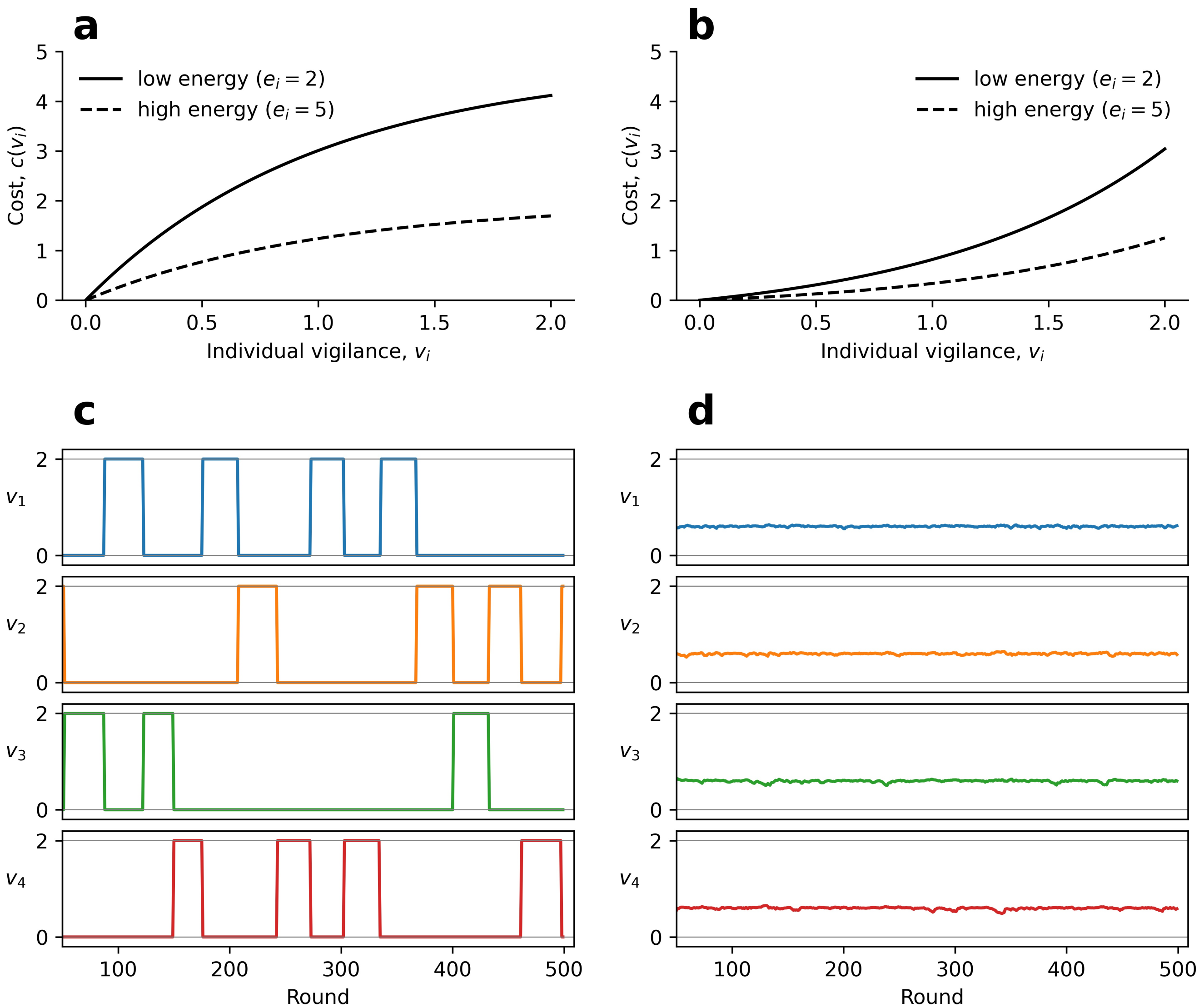

Fig. S3. Energy-dependent simulations with stochastic foraging for cooperative groups. a) Flattening (concave) costs with energetic scaling generate c) turn-taking of a sentinel role with maximum vigilance while others have zero vigilance. b) Steepening (convex) costs with energetic scaling generates d) no distinct roles with all individuals maintaining a similar level of vigilance with relatively small fluctuations.

this burden is spread approximately evenly through the group by turn-taking. One exception to this is when the metabolic cost is very low, $m = 0.1$, in which case the energetic state of sentinels does not decrease (in expectation) and so there is never any incentive to switch from a high vigilance role, so whichever individual takes on the sentinel role first stays there for the duration of the simulations. We also find zero vigilance when predation threat is very low, $r = 0.5$, matching the findings in the main paper. When cost scaling is very low, $C = 0.1$, the per-round equilibrium can be 3 or 4 sentinels depending on the individual energetic states, so that the mean number of sentinels is between 3 and 4.

When cost scaling is convex we overall find many-eyes behavior, with vigilance spread throughout the group and no turn-taking. The exception is when the predation threat is low, $r = 0.5$, where there is zero vigilance.

Table S1. Robustness checks for turn-taking simulation. For each set of simulation parameters (rows), we ran a single simulation with $10,000$ rounds and a burn-in of 50 rounds. We labeled an individual as vigilant in a period if their vigilance $v_i > 10^{-3}$. *Mean Individual Prop. Time Vigilant* is the proportion of periods spent vigilant, averaged across individuals; *SD* is the standard deviation of that fraction across individuals. *Mean # Vigilant/Round* is the mean number of vigilant individuals per round.

Cost Scaling	Varied	N	r	m	e_{max}	C	Mean Individual Prop. Time Vigilant	SD	Mean # Vigilant/Round
concave, $\alpha = -1$	baseline	4	5	0.3	10	10	0.249	0.018	0.994
concave, $\alpha = -1$	r	4	0.5	0.3	10	10	0.000	0.000	0.000
concave, $\alpha = -1$	r	4	50	0.3	10	10	0.496	0.009	1.985
concave, $\alpha = -1$	N	2	5	0.3	10	10	0.494	0.002	0.988
concave, $\alpha = -1$	N	8	5	0.3	10	10	0.125	0.022	0.998
concave, $\alpha = -1$	N	16	5	0.3	10	10	0.062	0.014	0.999
concave, $\alpha = -1$	m	4	5	0.1	10	10	0.250	0.433	1.000
concave, $\alpha = -1$	m	4	5	0.2	10	10	0.250	0.035	0.998
concave, $\alpha = -1$	m	4	5	0.5	10	10	0.247	0.019	0.987
concave, $\alpha = -1$	e_{max}	4	5	0.3	5	10	0.247	0.009	0.987
concave, $\alpha = -1$	e_{max}	4	5	0.3	20	10	0.494	0.009	1.976
concave, $\alpha = -1$	C	4	5	0.3	10	0.1	0.800	0.004	3.198
concave, $\alpha = -1$	C	4	5	0.3	10	1	0.496	0.009	1.985
convex, $\alpha = 1$	baseline	4	5	0.3	10	10	1.000	0.000	4.000
convex, $\alpha = 1$	r	4	0.5	0.3	10	10	0.000	0.000	0.000
convex, $\alpha = 1$	r	4	50	0.3	10	10	1.000	0.000	4.000
convex, $\alpha = 1$	N	2	5	0.3	10	10	1.000	0.000	2.000
convex, $\alpha = 1$	N	8	5	0.3	10	10	1.000	0.000	8.000
convex, $\alpha = 1$	N	16	5	0.3	10	10	1.000	0.000	15.996
convex, $\alpha = 1$	m	4	5	0.1	10	10	1.000	0.000	4.000
convex, $\alpha = 1$	m	4	5	0.2	10	10	1.000	0.000	4.000
convex, $\alpha = 1$	m	4	5	0.5	10	10	1.000	0.000	3.999
convex, $\alpha = 1$	e_{max}	4	5	0.3	5	10	1.000	0.000	3.999
convex, $\alpha = 1$	e_{max}	4	5	0.3	20	10	1.000	0.000	4.000
convex, $\alpha = 1$	C	4	5	0.3	10	0.1	1.000	0.000	4.000
convex, $\alpha = 1$	C	4	5	0.3	10	1	1.000	0.000	4.000

Turn-Taking Through Energetic Ordering

In the main paper we present simulations where the cost of vigilance scales as a function of the energetic state of individuals, which changes as a stochastic function of foraging efforts. These simulations generate turn-taking when cost scaling is concave and not when cost scaling is convex. Here, we present an alternative mechanism to generate turn-taking sentinels (Figure S4).

In these simulations, every individual in a group has the same cost function and this is not influenced by the energetic state. Instead, the energetic state determines the order in which individuals set their vigilance. Each round, all vigilances are first set to zero, then individuals choose their optimal vigilance in order of highest to lowest energetic state, and this itself loops until the group finds an equilibrium.

With concave costs, the individual with the highest energetic state takes on the sentinel role, while others have zero vigilance. As a result, the sentinel's energy drops (in expectation) while the rest of the group's energy rises. This continues until the energy of one of the non-sentinels rises above that of the sentinel. In the following round the non-sentinel takes on the sentinel role. As before, the sentinel loses energy while the non-sentinels gain energy, with the result that the sentinel role switches almost every round.

With convex costs, a distributed equilibrium is found each round. This is a symmetric equilibrium with all members having the same vigilance. The energetic states of the individuals will vary stochastically, changing the ordering of which individual sets their vigilance first. However, the same symmetric equilibrium is always found, so that the ordering makes no difference to the vigilances.

The simulations of turn-taking in the main paper were based on a scaling of costs based on energetic state, and vigilances persist from round to round. This generates a form of hysteresis where sentinels stay in a high vigilance role until their energetic

state drops to a point where they would prefer to forage than be vigilant, and then are replaced with another member of the group with a higher energetic state. In the simulations here, with energetic ordering, the turn-taking is much more erratic, with the sentinel role changing almost every round.

References

1. SH Strogatz, *Nonlinear dynamics and chaos: with applications to physics, biology, chemistry, and engineering (studies in nonlinearity)*. (Westview Press) Vol. 1, (2001).
2. J Maynard Smith, GR Price, The logic of animal conflict. *Nature* **246**, 15–18 (1973).
3. J Maynard Smith, Evolution and the theory of games in *Did Darwin get it right? Essays on games, sex and evolution.* (Springer), pp. 202–215 (1982).
4. PA Bednekoff, Mutualism among safe, selfish sentinels: a dynamic game. *The Am. Nat.* **150**, 373–392 (1997).

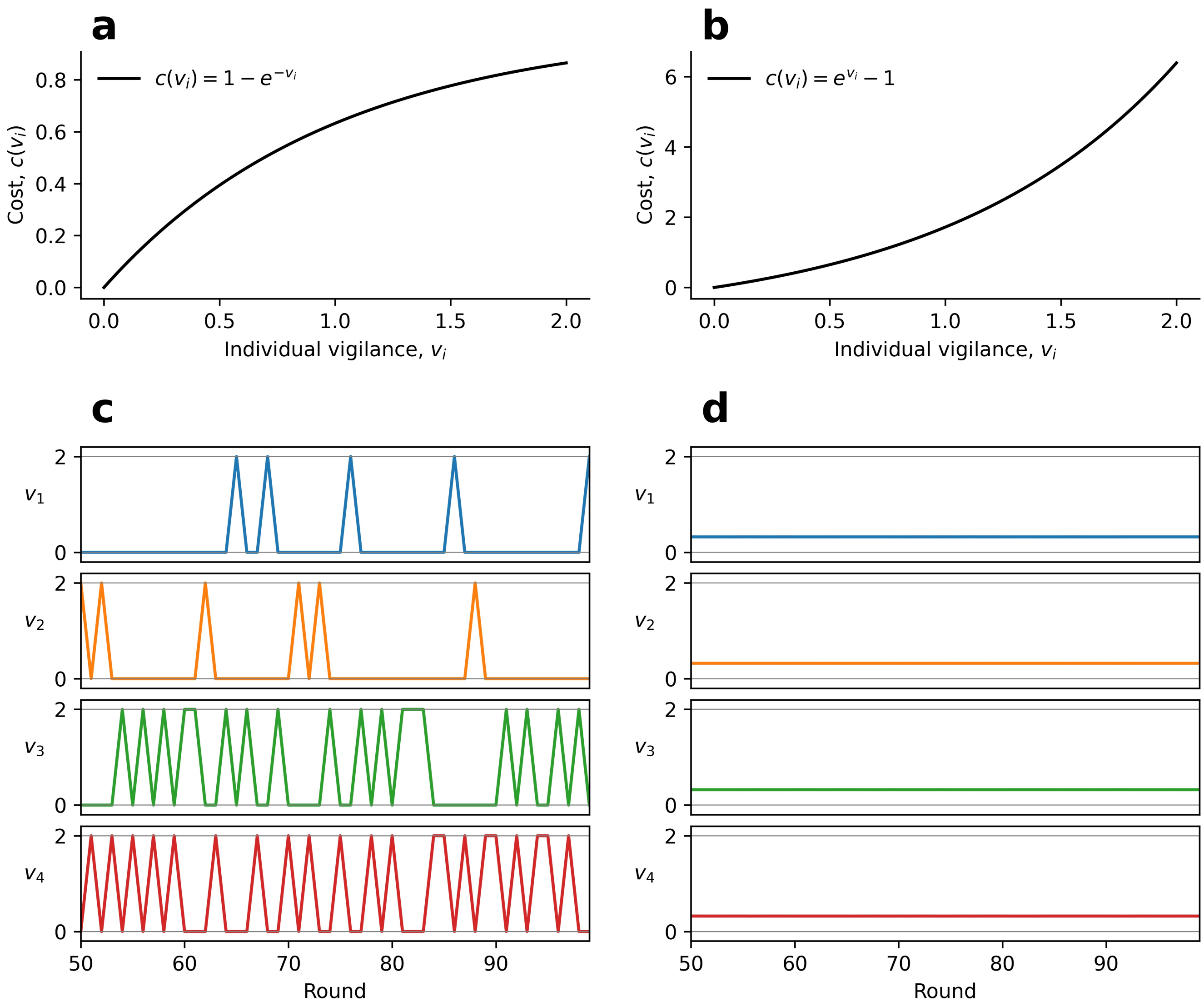

Fig. S4. Energy-ordered simulations with stochastic foraging for selfish groups. a) Flattening (concave) costs with energetic ordering generate c) frequent turn-taking of a sentinel role with maximum vigilance while others have zero vigilance. b) Steepening (convex) costs with energetic ordering generates d) no distinct roles with all individuals maintaining the same level of vigilance. To clearly show the dynamics, the x-axis is truncated to show rounds 50 to 100; this is a representative sample.
